# Parathyroid Auto-transplantation Mimicking Liposarcoma

**DOI:** 10.7759/cureus.5445

**Published:** 2019-08-20

**Authors:** Agnieszka Boron, John V Dennison, Chelsea B Dennison, Ivey Royall, Kurt Scherer

**Affiliations:** 1 Internal Medicine, University of Central Florida College of Medicine, Orlando, USA; 2 Radiology, Florida Hospital, Orlando, USA; 3 Radiology - GME, AdventHealth Orlando, Orlando, USA

**Keywords:** liposarcoma, parathyroid autotransplantation, hyperparathyroidism

## Abstract

Parathyroid autotransplantation is an increasingly common procedure given the increasing rate of hyperparathyroidism. However, post-autotransplantation imaging is not commonly performed and the imaging findings can mimic liposarcoma. Therefore, radiologists should be aware of the imaging characteristics of parathyroid autotransplantation. Here we discuss the CT and Tc99m-Sestamibi 4D-CT findings of parathyroid autotransplantation. We will also discuss the pathophysiology of liposarcoma and present the gross histological findings seen on pathology.

## Introduction

Implantation of parathyroid tissue into the forearm after a total parathyroidectomy or thyroidectomy is well described [1-3]. Although the sternocleidomastoid muscle is the preferred location for autotransplantation, the forearm serves as a useful reservoir due to our ability to remove the graft if needed [2]. After parathyroid tissue is removed, intraoperative parathyroid hormone levels are checked to ensure a decline and part of the tissue is subsequently either cryopreserved or immediately autotransplanted [1]. Imaging of the autotransplanted tissue is typically not done, and no research exists on how parathyroid tissue in the forearm appears on a computed tomography (CT) scan. The case of an African-American male who underwent autotransplantation of parathyroid tissue into his right forearm, which was later interpreted to be a lipomatous mass on CT, is presented.

## Case presentation

**Figure 1:**
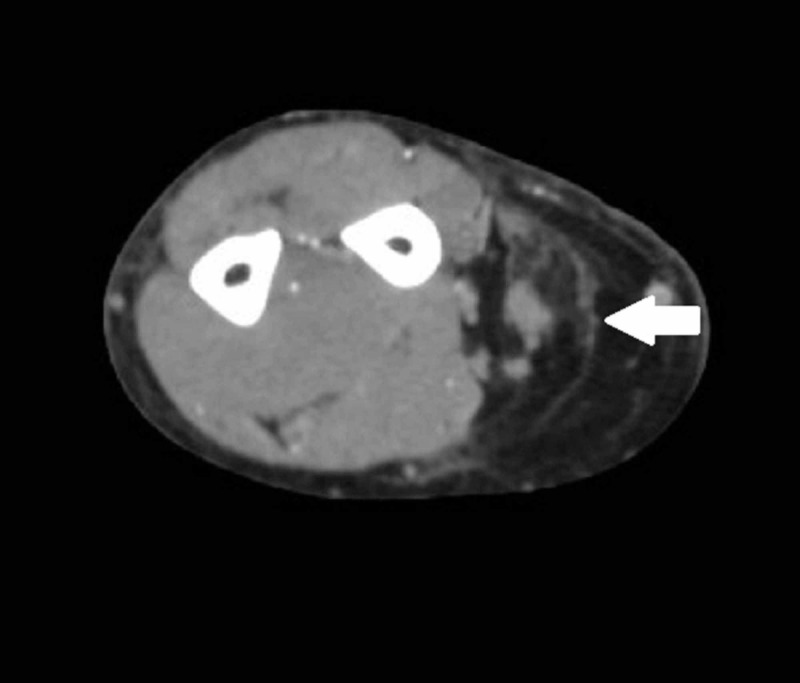
A 56-year-old male with a right forearm fat containing mass (thick white arrow) demonstrated on axial CT image, concerning for an atypical lipomatous tumor or liposarcoma.

**Figure 2:**
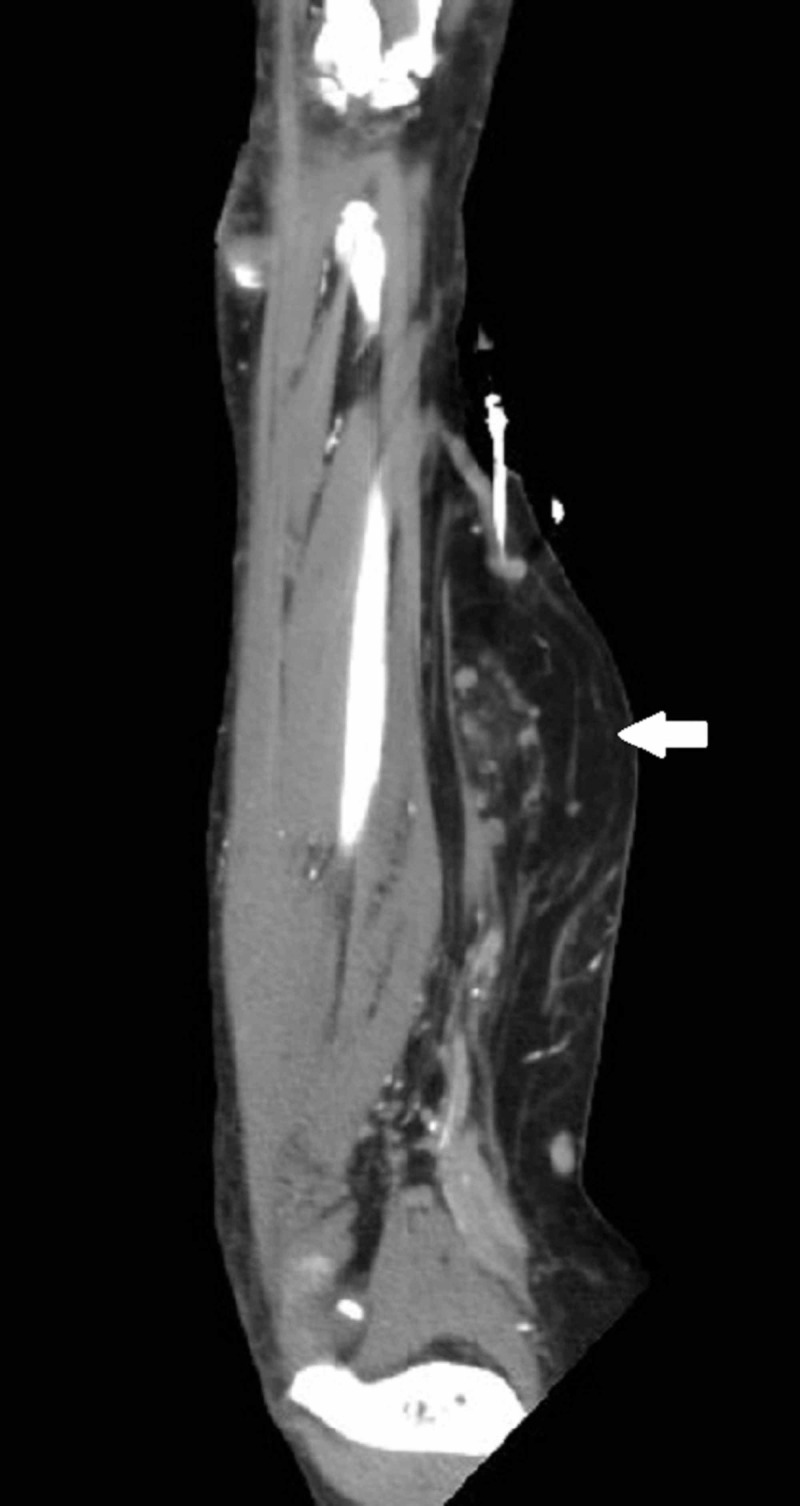
Coronal reformatted CT also showing a right forearm mass (thick white arrow) concerning for atypical lipomatous tumor or liposarcoma.

A 56-year-old male with a past medical history of end-stage renal disease on hemodialysis, congestive heart failure, groove pancreatitis, chronic atrial fibrillation, valvular heart disease, and obesity, who was status post four gland parathyroidectomy for hyperparathyroidism seven years prior presented to the hospital for chronic bilateral lower extremity nonhealing wounds which had been worsening. He was found to have a parathyroid hormone level of over 3,200 pg/mL (normal range 10-65 pg/mL) as well as a soft right forearm mass located at the site of previous autotransplantation. The mass was further characterized with a CT scan of his right forearm under the indication “distal forearm tumor”. The CT scan showed a fat containing mass at the lateral aspect of his forearm measuring 4.6 x .9 x 5 cm with soft tissue nodularity suspicious for an atypical lipomatous tumor or liposarcoma (Figures 1, 2).

**Figure 3:**
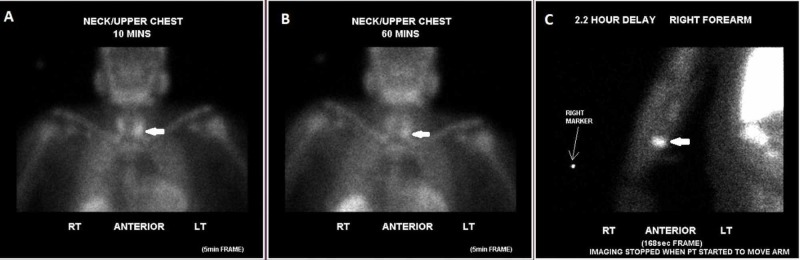
Technetium-99m Sestamibi scan demonstrates increased uptake posterior and inferior to the left lobe of the thyroid gland at both 10 minute (A) and 60 minute (B) as well as focal increased uptake within the mid-right forearm on a two-hour delayed image (C) consistent with residual parathyroid tissue in the neck and ectopic thyroid tissue (thick white arrow) in the right forearm.

**Figure 4:**
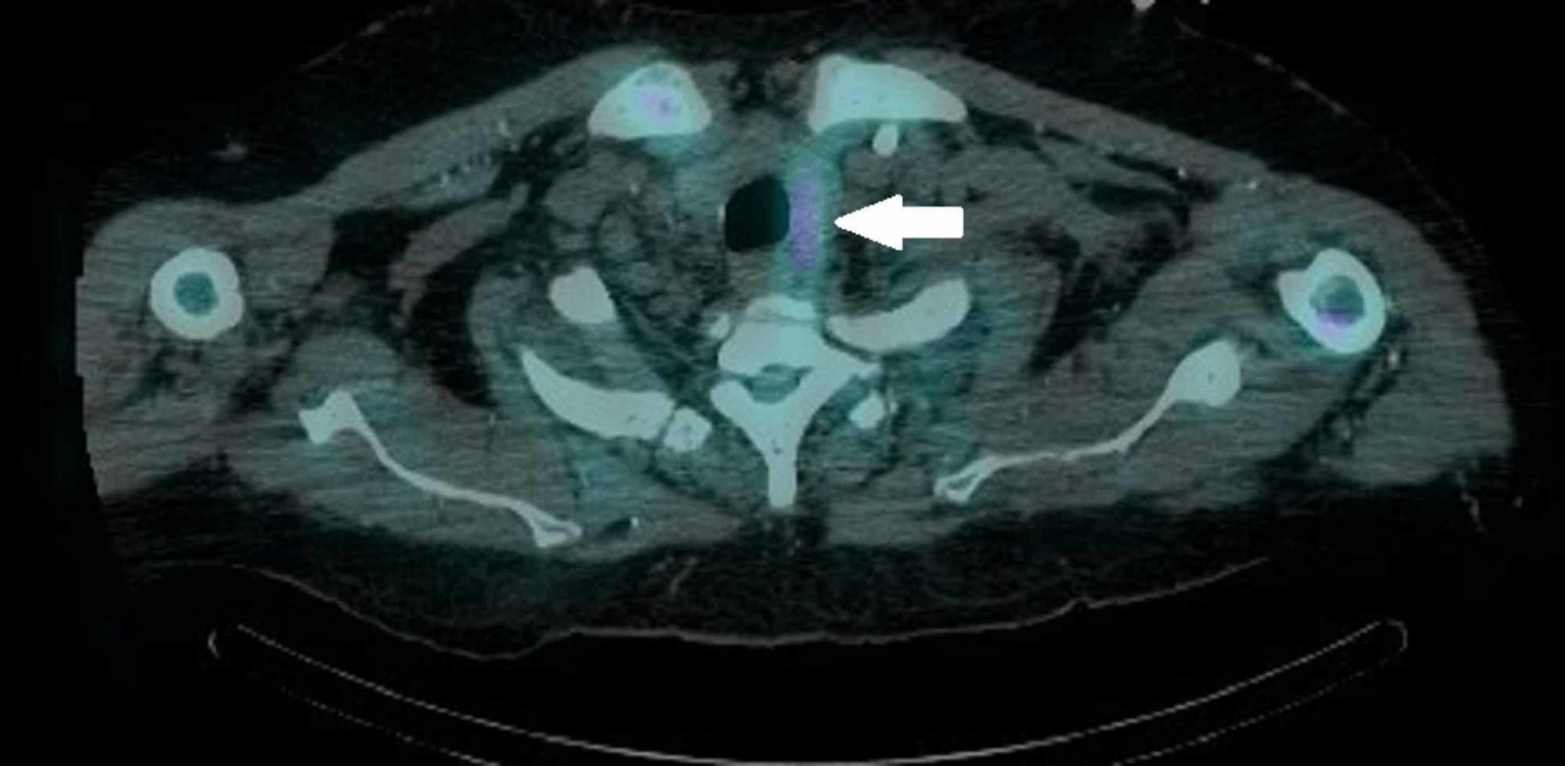
4D-CT scan of the lower neck demonstrates a 1.5 x 2 x 1.5 cm nodule with increased radiotracer uptake posterior to the lower pole of the left thyroid gland consistent with residual parathyroid tissue or parathyroid adenoma (thick white arrow).

Follow-up imaging with a nuclear medicine Technetium-99m Sestamibi scan showed retained activity posterior and inferior to the left lobe of the thyroid gland and focal increased activity at the right forearm on a two-hour delayed image (Figure 3). A 4D-CT scan of the parathyroid showed a 1.5 x 2 x 1.5 cm nodule posterior to the lower pole of the left lobe of the thyroid gland, suggestive of a parathyroid adenoma or thyroid nodule (Figure 4).

**Figure 5:**
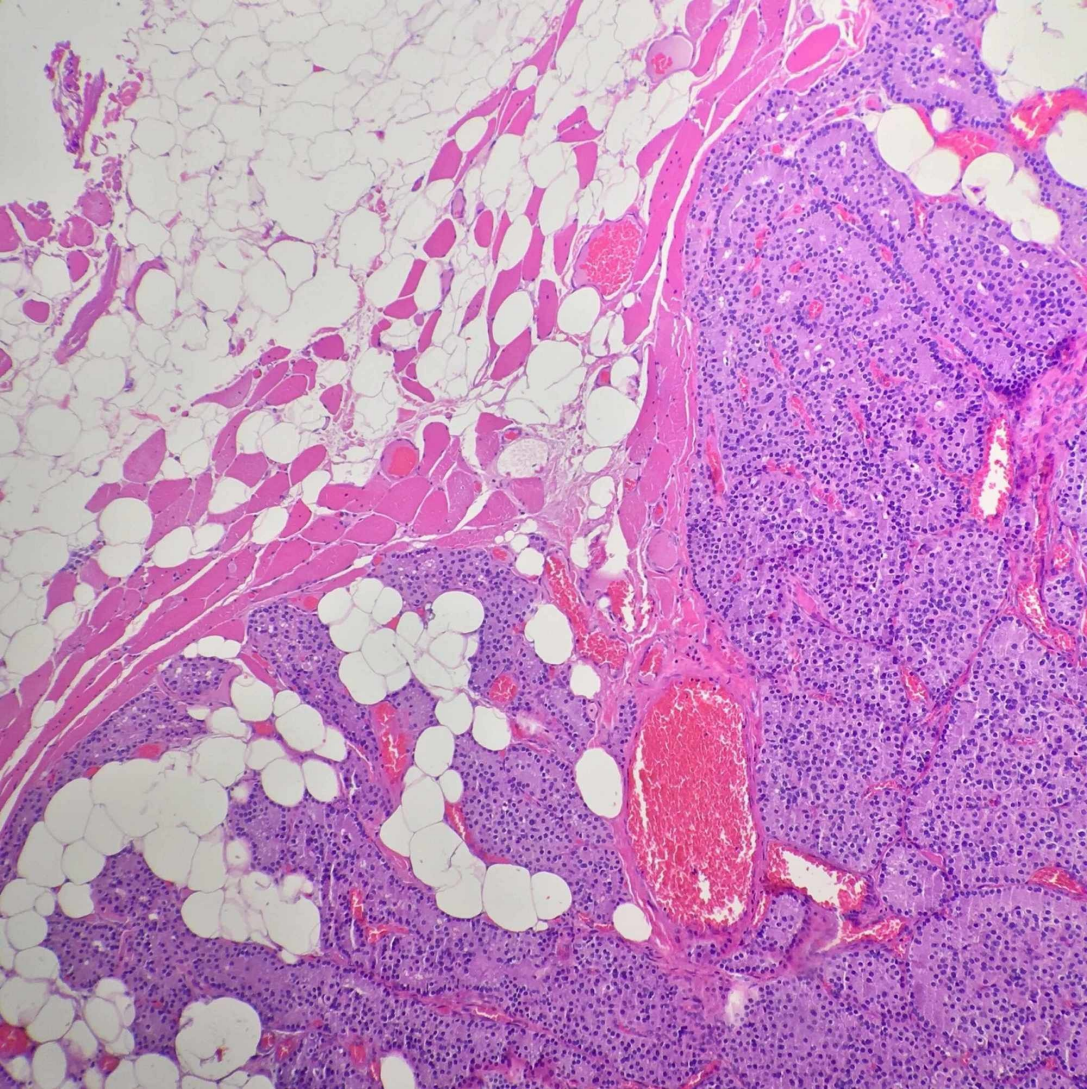
Low magnification H&E stain of the forearm mass showing nodules of hyperplastic parathyroid tissue with intervening fibro-adipose tissue and skeletal muscle. H&E: Hematoxylin and eosin

**Figure 6:**
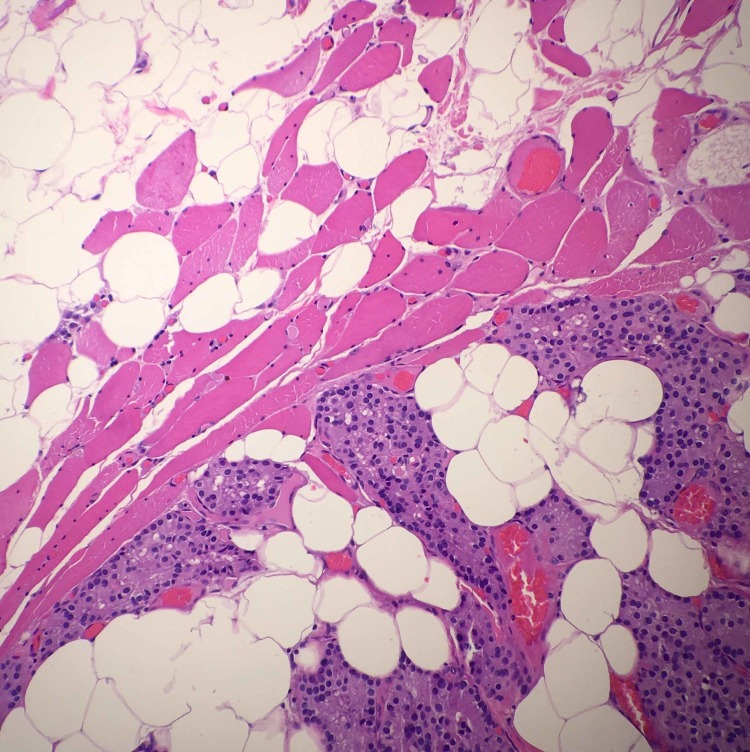
High magnification H&E stain of the same mass also showing parathyroid tissue. H&E: Hematoxylin and eosin

Because of his markedly elevated parathyroid hormone (PTH) level and concern that the mass on his right forearm may be a lipomatous tumor due to the CT scan interpretation, the decision was made to resect the mass. Upon resection, the operative report stated that the mass did have the appearance of possible lipomatous features given that it had a different characteristic than the surrounding subcutaneous fat. The surgical pathology report came back as nodules of hyperplastic parathyroid tissue involving fibroadipose tissue and skeletal muscle (Figures 5, 6).

**Figure 7:**
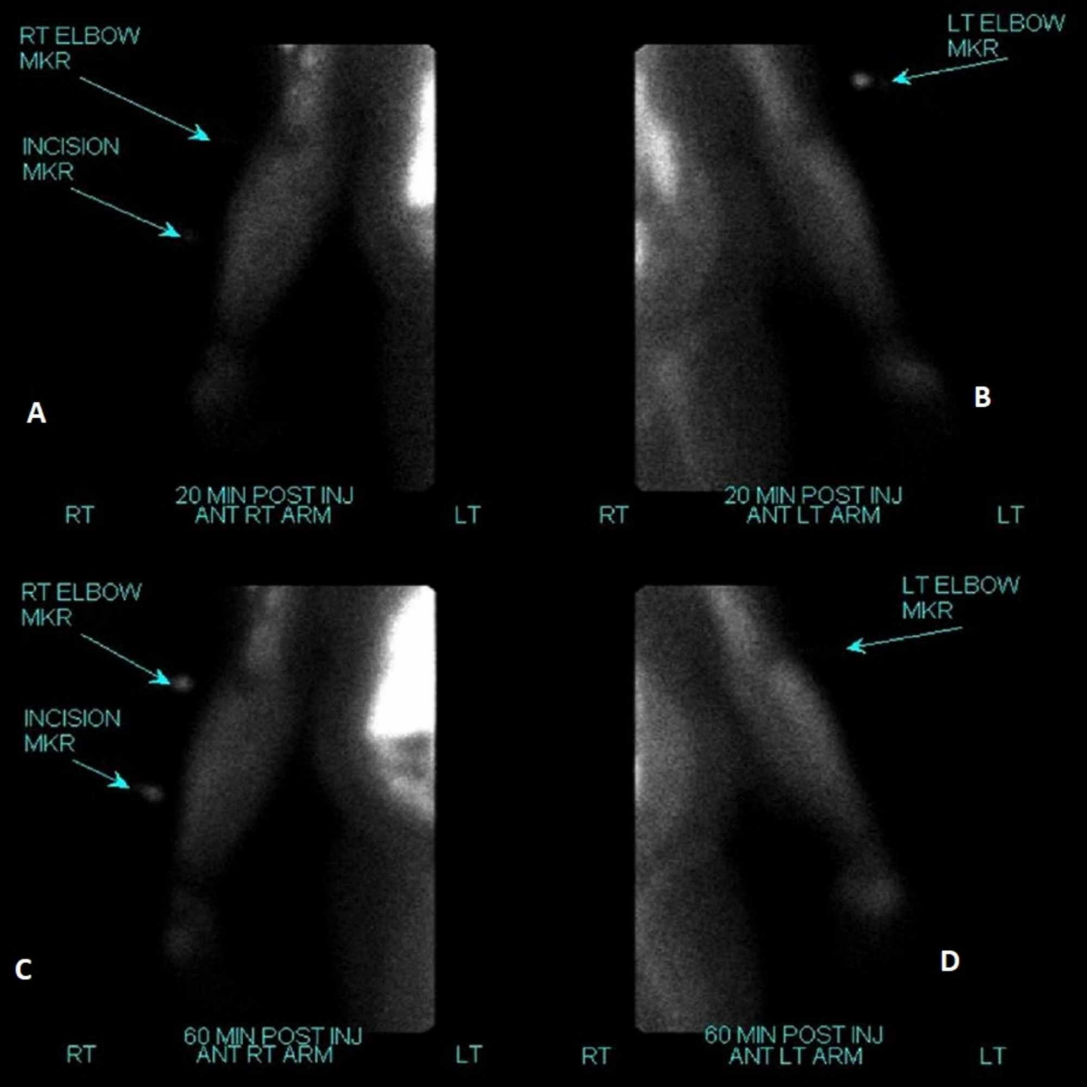
After surgical resection of the right forearm mass, a repeat Technetium-99m Sestamibi scan demonstrated no focal areas of uptake to suggest residual parathyroid tissue after resection (A & C). Left forearm is shown for comparison (B & D).

A repeat Technetium-99m Sestamibi study performed three days later showed no uptake at 20- and 60-minute delayed imaging to suggest residual parathyroid tissue (Figure 7). Intraoperatively his PTH level was still markedly elevated and he eventually underwent additional parathyroid tissue removal with a left thyroid lobectomy several months later.

## Discussion

Parathyroid tissue during a total parathyroidectomy can be preserved in situ, autotransplanted to the brachioradialis or sternocleidomastoid muscle, or allotransplanted from a donor [4]. There is no widely accepted surgical technique. Monchik and Cotton showed success with using frozen section biopsy after gland removal to confirm excision of parathyroid tissue, then monitoring for a drop in the intraoperative parathyroid hormone level, followed by transplanting the least nodular and smallest gland to the forearm in six 1 x 2 mm pieces [5]. Post-operatively, CT scan imaging has not been reported. With the increasing incidence of hyperparathyroidism and different parathyroid preservation techniques it is pertinent that radiologists are aware of the variations in appearance [4,6]. Liposarcomas are tumors of adipocyte origin [7]. They are categorized into atypical lipomatous/well-differentiated tumors, dedifferentiated, myxoid, and pleomorphic [8]. Lipomas and liposarcomas can have similar features on magnetic resonance imaging (MRI). In one retrospective study of 87 patients, adipocyte masses were more likely to be liposarcomas if they were diagnosed at over 60 years of age, were over 10 cm, located in the patient's lower limbs, and had non-fatty areas. Liposarcomas exhibited more septations and increased signal intensity on MR when compared to lipomas, although lipomas also had these features [9]. CT scanning can also aid in the diagnosis of liposarcomas. Atypical liposarcomas appear as fat-like densities with strip-like septations that are completely encapsulated [10]. Definitive diagnosis on tumor subtype is still reached by biopsy and molecular testing [11].

## Conclusions

Parathyroid autotransplantation is a common procedure done after parathyroidectomy in the setting of hyperparathyroidism. Lack of imaging of autotransplantation can be challenging in that its appearance can mimic a lipomatous malignancy such as liposarcoma, with the diagnosis necessitating biopsy for the final diagnosis. In the setting of hyperparathyroidism after parathyroidectomy, nuclear medicine studies such as Technetium-99m Sestamibi scan may be helpful in identifying the location of the transplanted parathyroid gland.

## References

[1] Brunt LM, Sicard GA (1990). Current status of parathyroid autotransplantation. Semin Surg Oncol.

[2] Cavallaro G, Iorio O, Centanni M (2015). Parathyroid reimplantation in forearm subcutaneous tissue during thyroidectomy: a simple and effective way to avoid hypoparathyroidism. World J Surg.

[3] Krausz MM, Ashkenazi I, Alfici R (2017). Parathyroid autotransplantation in adults and children. (Article in Hebrew). Harefuah.

[4] Barczynski M, Golkowski F, Nawrot I (2017). Parathyroid transplantation in thyroid surgery. Gland Surg.

[5] Monchik JM, Cotton TM (2017). Technique for subcutaneous forearm transplantation of autologous parathyroid tissue. Surgery.

[6] Yeh MW, Ituarte PH, Zhou HC (2013). Incidence and prevalence of primary hyperparathyroidism in a racially mixed population. J Clin Endocrinol Metab.

[7] Jo VY, Doyle LA (2016). Refinements in sarcoma classification in the current 2013 World Health Organization Classification of tumours of soft tissue and bone. Surg Oncol Clin N Am.

[8] Muratori F, Frenos F, Bettini L (2018). Liposarcoma: clinico-pathological analysis, prognostic factors and survival in a series of 307 patients treated at a single institution. J Orthop Sci.

[9] Brisson M, Kashima T, Delaney D (2013). MRI characteristics of lipoma and atypical lipomatous tumor/well-differentiated liposarcoma: retrospective comparison with histology and MDM2 gene amplification. Skeletal Radiol.

[10] Lu J, Qin Q, Zhan LL (2014). Computed tomography manifestations of histologic subtypes of retroperitoneal liposarcoma. Asian Pac J Cancer Prev.

[11] Alaggio R, Coffin CM, Weiss SW, Bridge J, Issakov J, Oliveira A, Folpe A (2009). Liposarcomas in young patients: a study of 82 cases occurring in patients younger than 22 years of age. Am J Surg Pathol.

